# Female Mating Frequency and Reproductive Fitness in the Willow Leaf Beetle (Coleoptera: Chrysomelidae)

**DOI:** 10.1093/jisesa/iez116

**Published:** 2019-11-29

**Authors:** Lvquan Zhao, Ying Qiu, Xiaodi Shi, Wei Wang, Shouzhu Liu

**Affiliations:** 1 Co-innovation Center for Sustainable Forestry in Southern China, College of Forestry, Nanjing Forestry University, Nanjing 210037, China; 2 School of Agriculture, Liaocheng University, Liaocheng 252059, China

**Keywords:** multiple mating, fecundity, interval mating, egg hatchability, longevity

## Abstract

Multiple mating in females is common in nature but may involve fitness costs. Adult females and males of the beetle *Plagiodera versicolora* Laicharting can mate multiple times. We studied the effect of mating frequency and mating pattern (time interval between matings) on female reproductive fitness by measuring fecundity, hatching probability, and female longevity. Fecundity and longevity were similar in single- and double-mated (two matings separated by a 7 d interval) females. However, two and three successive matings and three matings separated by two 7 d interval had a significant negative effect on the lifetime fecundity and longevity of females. Multiple mating had a positive effect on egg hatching, and two matings sufficed to fertilize the full egg load. These results indicate that the two matings separated by a 7 d interval are optimum for reproductive fitness in female *P. versicolora*. Suboptimal mating frequency (successive mating or an excessive number of matings) exacts a physiological cost that shortens the female life span and reduces fecundity.

Multiple mating (polyandry) by females is common in insects, even when males provide no material benefits such as food or parental care (Simmons 2005). Multiple mating by females in such circumstances is difficult to explain because mating carries associated costs (Barbosa et al. 2012). In addition to the energy and time engaged in mating, multiple mated females may have increased risks of predation, transmission of parasites, physical damage, and/or disease transmission (Kawagoe et al. 2001). They may also have a shorter life span as well as reduced time for feeding and oviposition (Keller and Reeve 1995). Thus, these costs have a negative influence on female fitness (Arnqvist and Nilsson 2000). Understanding why multiple mating by females is standard, rather than an exception, in the absence of material benefits remains challenging (Firman and Simmons 2012, Sardell et al. 2012).

Multiple mating has been extensively studied in the context of understanding the evolution of mating strategies (Simmons 2005). In theory, multiple mating should only be adaptive if the mating costs are offset by benefits that enhance female fitness (Hosken and Stockley 2003). The hypotheses used to explain the adaptive value of multiple mating can be divided into two general classes: nongenetic benefits (direct/first generation) (Arnqvist and Nilsson 2000) and genetic benefits (indirect/second genetation) (Jennions and Petrie 2000). Sperm limitation may affect female sex allocation and offspring production (Wang et al. 2018, Chirault et al. 2019). If a female receives insufficient sperm from one mating, then additional matings will help ensure that all eggs are fertilized and provide a fecundity benefit (Olsson and Shine 1997). A direct benefit of multiple mating may be increased fecundity (Arnqvist and Nilsson 2000). *Cnaphalocrocis medinalis* females mating two or three times laid significantly more eggs with significantly higher hatchability than females mating once (Kawazu et al. 2014).

Indirect benefits (heritable and genetic benefits to the next generation) are associated with postmating sexual selection mechanisms that are promoted by mating with multiple, genetically different, males (Yasui 1998). Postmating sexual selection can select for compatible genes, thus reducing inbreeding and result in offspring with greater fitness (Zeh and Zeh 2003, Michalczyk et al. 2011, Firman and Simmons 2012). Alternatively, postmating sexual selection may also favor males, with competitive ejaculates, to sire more offspring (Yasui 1998). Indirect benefits may also increase offspring attractiveness and viability (Gowaty et al. 2010).

Despite considerable empirical evidence that females benefit from multiple mating, some species show a different response. In *Drosophila melanogaster* and *Colaphellus bowringi*, multiple mating decreases female reproductive fitness (Brown et al. 2004, Liu et al. 2013). Most studies evaluating the costs and benefits of multiple mating use ‘successive mating’ model (Arnqvist and Nilsson 2000). However, in females that are capable of multiple mating, the mating pattern can include successive multiple mating (mating with two or more males in succession during 1 d) or interval multiple mating (mating with two or more males during their entire life span). Interval multiple mating and successive multiple mating may affect reproductive fitness in different ways. It is unclear whether interval multiple mating will bring female more reproductive fitness than successive multiple mating.

The willow leaf beetle *Plagiodera versicolora* Laicharting is widely distributed in Asia, Europe, and North Africa. It feeds on the leaves and shoots of willows and poplar species (Ishihara and Suzue 2011). The adults lay egg clutches on willow leaves and the number of eggs per clutch varies from 1 to 37 (average = 15) (Ishihara and Hayashi 2000). Young larvae feed in groups of related individuals, but mature (third instar) larvae are typically solitary (Ishihara and Hayashi 2000). In Nanjing city, China, *P. versicolora* has eight to nine generations per year, and new adults emerge from April to November (L.Z., personal observation). Females can mate with another male immediately after completion their first mating and they can mate multiple times during their lifetime (L.Z., personal observation).

Aiming to test the above possibilities and further our understanding on polyandry, in the present study, we compared the reproductive fitness of *P. versicolora* females with interval multiple mating and successive multiple mating using the same mating frequency. The results of this study will increase our understanding of the adaptive significance and evolution of multiple mating in this species.

## Materials and Methods

### Insect Rearing

Larvae of *P. versicolora* were collected from their host plant, *Salix babylonica*, in April 2019 on the campus of Nanjing Forestry University, Nanjing City, Jiangsu Province. The beetles were reared on *S. babylonica* in transparent plastic boxes (5 cm diam. × 4 cm high). Leaves were replenished daily until the larvae reached the prepupal state. After pupation, the pupae were transferred to a separate box for adult emergence. After emergence, the adults were reared in groups for oviposition. The females laid eggs on *S. babylonica* leaves that were changed daily. After hatching, we randomly selected 15–20 larvae, transferred them to a new box with fresh *S. babylonica* leaves, and reared them to pupation. On the day of adult emergence, the adults were transferred and reared individually in separate containers prior to use in experiments. All experiments were conducted in chambers at a constant 26 ± 1°C and L14: D 10 photoperiod. During multiple mating experiments, we supplied leaves from the same *Salix* clone for all insects because the host-plant clone may affect the growth and reproduction of *P. versicolora* (Egusa et al. 2008).

### Reproductive Fitness of Females Mated Multiply in Succession

Preliminary studies showed that the preoviposition period of *P. versicolora* females is about 7 d. Therefore, the mating experiments were initiated on the 7th day after adult emergence. The 7 d females were divided into three treatment groups. In Group 1 (1 mating), a virgin female was mated once with a 7 d old virgin male. After a female was transferred into the container, the male moved close to the female and made contact with the female, which was accompanied by the male occasionally tapping the female with his antennae. Subsequently, the male climbed onto the back of the female and inserted his genital into female genital and then pulled out his genital after mating. We scored a successful mating as one in which the male genital remained in the females for at least 20 min. The male was removed after a successful mating and the female was reared alone. In Group 2 (two successive matings), each virgin female was allowed to mate with a 7 d old virgin male; the male was removed after successful mating, then different 7 d old virgin male was transferred into the same container to mate with the female. After the second successful mating, the male was removed and the female was reared alone. In Group 3 (three successive matings), each female was allowed to mate with virgin males three times as performed in Group 2. The males were removed after mating, and the female was reared alone. The control group was 30 females that remained unmated and were individually reared in plastic plates.

### Reproductive Fitness of Females Mated Multiply with Time Intervals

Similar to the above experiments, we divided 7 d old females into three groups. For Group 1 (1 mating), a virgin female was paired with a 7 d old virgin male. Then the male was removed and the female was reared individually. In Group 2 (two matings separated by a 7 d interval), a virgin female was mated with a male. The male was then removed, and the female was reared individually. Seven days later, a new 7 d old virgin male was transferred to the same container to mate with the mated female, and after the second successful mating, the male was removed, and the female was reared individually. In Group 3 (three matings separated by two 7 d interval), a virgin female was assigned to mate with a 7 d old virgin male sequentially three times at 7 d, 14 d, and 21 d, respectively, after adult emergence as performed in Group 2. The male was removed after each successful mating, and the female was reared individually in every mating.

### Measurements of Reproductive Fitness

We quantified three parameters of female reproductive fitness: fecundity, egg hatching rate, and female longevity. Females laid eggs in egg clutches. Once a female began laying eggs, the egg clutches of each mated female was transferred, along with the willow leaves, to a new Petri dish with fresh filter paper every day for egg counts and egg hatching per clutch over her life span. We also recorded the time from pairing to successful mating, the mating duration (the time from male inserting his genital into the female genital to retracting his genital) and the egg-laying duration (the time from first oviposition to last oviposition) in the mating schedule given above.

### Statistical Analysis

Time from pairing to successful mating, mating duration, number of egg clutches, number of eggs per egg clutch, and egg hatching were analyzed by one-way analysis of variance (ANOVA). Differences in fecundity were analyzed by ANCOVA with body weight of the female as the covariate. The means were separated using Fisher’s least significant difference (LSD) test when the *F* values were significant (*P* < 0.05). All statistical analyses were performed using SPSS 22.0 (IBM Inc., New York).

## Results

### Time from Pairing to Successful Mating and Mating Duration

Females and males initiated successful mating within 6 min when females were assigned to mate with males on any mating schedule; the times from pairing to successful mating in the different treatments were similar (ANOVA, *F* = 1.7; df = 10, 329; *P* = 0.08, Table 1). However, mating status had an obvious effect on mating duration among the five treatments (ANOVA, *F* = 22.2; df = 10, 329; *P* <0.001, Table 1). The mating duration of females assigned to mate with a male twice and three times was longer than the duration of females mating only once (LSD test, *P* < 0.05 in all cases, Table 1). The mating duration of females assigned to successively mate with a male twice and three times was longer than that of females mating with males twice and three times on an interval (LSD test, *P* < 0.05 in all cases, Table 1). The duration of three successive matings was the longest of all treatments (Table 1).

**Table 1.** Effect of mating pattern on the time from pairing to successful mating and mating duration of *Plagiodera versicolora*

Mating pattern	Mating number	Time from pairing to successful mating (min)	Mating duration (min)
Single mating	First time	3.1 ± 0.6 a	44.6 ± 4.3 a
Two successive matings	First time	2.6 ± 0.5 a	49.1 ± 3.1 a
	Second time	2.8 ± 0.4 a	66.7 ± 3.7 b
Two matings separated by a 7 d interval	First time	4.6 ± 1.2 a	45.9 ± 4.1 a
	Second time	2.7 ± 0.5 a	57.5 ± 4.2 c
Three successive matings	First time	5.1 ± 1.1 a	47.5 ± 3.9 a
	Second time	4.6 ± 0.8 a	68.9 ± 2.8 b
	Third time	4.7 ± 0.8 a	87.8 ± 3.8 d
Three matings separated by two 7 d interval	First time	5.7 ± 1.4 a	44.4 ± 3.1 a
	Second time	2.4 ± 0.4 a	61.7 ± 5.1 c
	Third time	4.6 ± 0.8 a	60.5 ± 4.4 c

Different letters after means within a column indicate significant differences between the given treatments (ANOVA, *P* < 0.05).

### Female Longevity

**Fig. 1.**
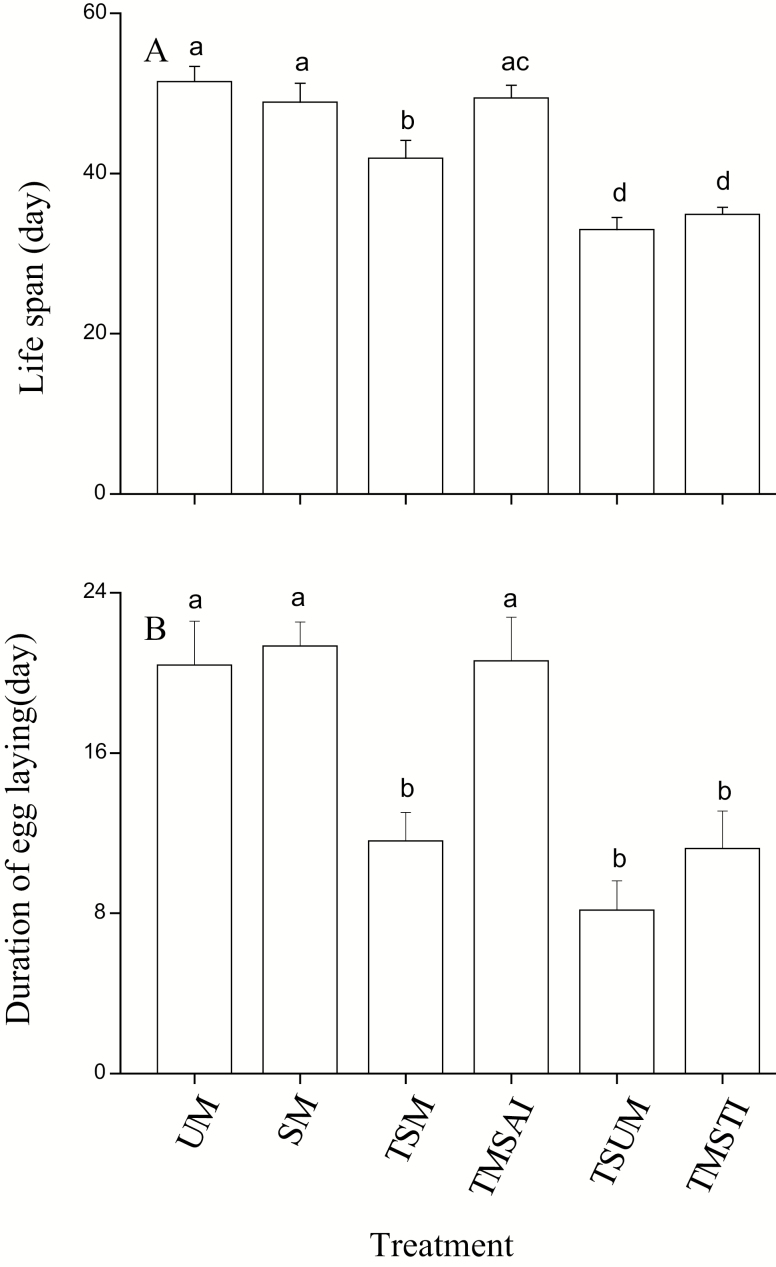
Effect of mating status on the life span (A) and duration of egg-laying (B). Error bars represent the SE. Bars with different letters are significantly different from each other at *P* < 0.05 by Fisher’s protected least significant test. UM: unmated females; SM: single mating; TSM: two successive matings; TMSAI: two matings separated by a 7 d interval; TSUM: three successive matings; TMSTI: three matings separated by two 7 d interval.

There was a significant difference in female longevity among the treatments (ANOVA, *F* = 21.8; df = 5, 172; *P* < 0.001, Fig. 1A). Virgin females lived an average of 54.5 ± 1.9 d, which was similar to females that mated only once (48.8 ± 2.3 d) and two matings separated by a 7 d interval (49.4 ± 1.6 d) (LSD test, *P* = 0.97 and 0.95, Fig. 1A). These longevity values were longer than those of two successive mating females, three successive mating females and three matings separated by two 7 d interval females (LSD test, *P* < 0.05 in all cases, Fig. 1A). Females that mated with males twice also lived longer than females mated with males three times (LSD test, *P* < 0.05 in all cases, Fig. 1A). The longevity of two matings separated by a 7 d interval females was significantly longer than that of two successive mating females (LSD test, *P* = 0.022, Fig. 1A).

### Duration of the Egg-Laying Period

Mating status had a significant effect on the duration of the egg-laying period (ANOVA, *F* = 8.2; df = 5, 163; *P* < 0.001, Fig. 1B). The egg-laying duration of once mated females was 21.9 ± 2.6 d, which was significantly longer than that of two successive mating females, three successive mating females and three matings separated by two 7 d interval females (LSD test, *P* < 0.001 in all cases, Fig. 1B), although they did not lay eggs longer than the virgin females (LSD test, *P* = 0.15, Fig. 1B). The egg laying duration of two matings separated by a 7 d interval females was similar to the single mated females and virgin females (LSD test, *P* = 0.93 and 0.84, Fig. 1B), and it was significantly longer than that of the two successive mating females, three successive mating females and three matings separated by two 7 d interval females (LSD test, *P* < 0.001 in all cases, Fig. 1B).

### Fecundity

**Fig. 2.**
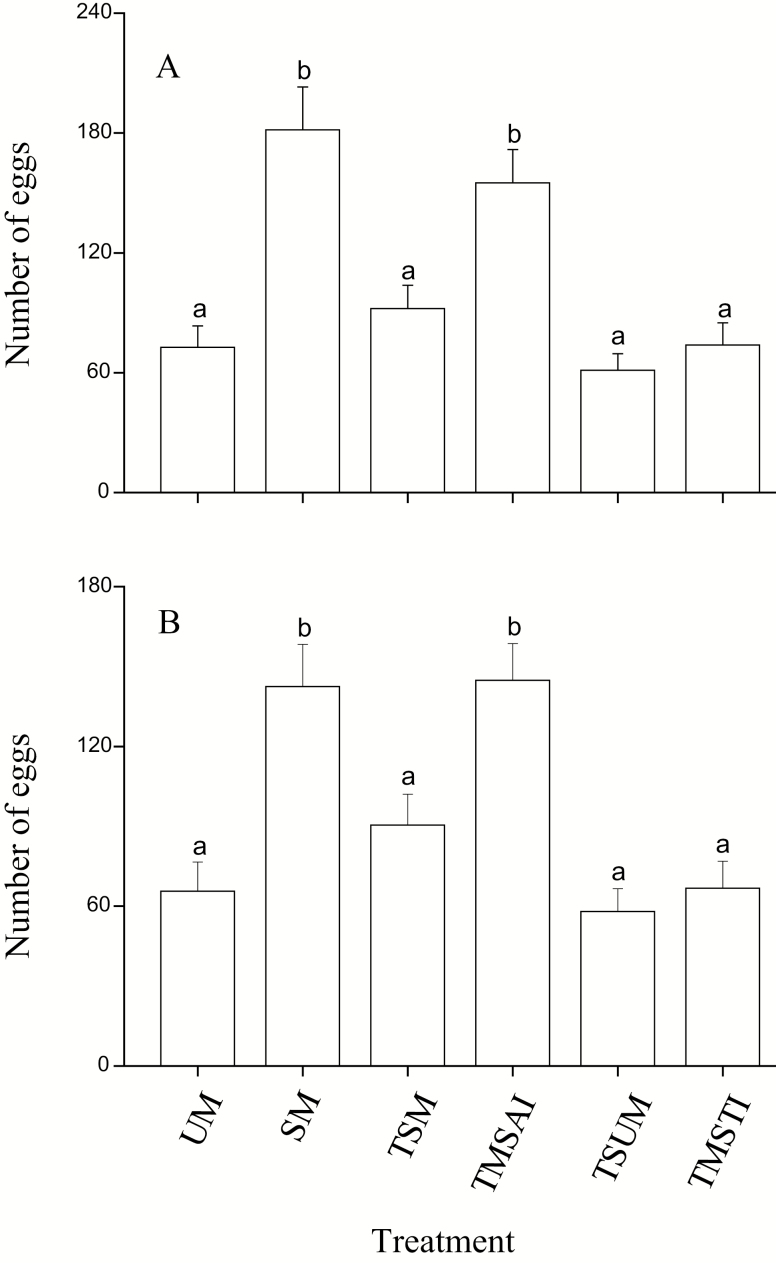
Comparison of the lifetime number of eggs (A) and eggs during the period of 25 d after adult emergence (B). Error bars represent the SE. Bars with different letters are significantly different from each other at *P* < 0.05 by Fisher’s protected least significant test. UM: unmated females; SM: single mating; TSM: two successive matings; TMSAI: two matings separated by a 7 d interval; TSUM: three successive matings; TMSTI: three matings separated by two 7 d interval.

Mating status significantly influenced the number of lifetime eggs (ANCOVA with body weight of the female as a covariate, *F* = 14.82, df = 5, 162, *P* < 0.001, Fig. 2A). Females mated only once had greater fecundity than virgin females and females with two successive matings, three successive matings and three matings separated by two 7 d interval (LSD test, *P* < 0.001 in all cases, Fig. 2A). There was no obvious difference in fecundity between females mated only once and two matings separated by a 7 d interval (LSD test, *P* = 0.75, Fig. 2A). Fecundity of the two successive mating females, three successive mating females and three matings separated by two 7 d interval females was similar to the fecundity of virgin females (LSD test, *P* = 0.92, 0.99, and 1.00, Fig. 2A).

To clarify that the difference in fecundity between the treatments was caused by decreased capacity of egg-laying rather than shortened duration of the egg-laying period, we compared the fecundity of treatments during the same 25 d oviposition period after adult emergence. Similar to the number of eggs laid during their lifetime, the fecundity during this 25 d period was also significantly influenced by mating status (ANCOVA with body weight of the female as a covariate, *F* = 10.86, df = 5, 162, *P* < 0.001, Fig. 2B). Females mated only once and two matings separated by a 7 d interval produced similar numbers of eggs (LSD test, *P* = 0.89, Fig. 2B), but they laid significantly more eggs than two successive mating females, three successive mating females, and three matings separated by two 7 d interval females (LSD test, *P* < 0.001 in all cases, Fig. 2B). There was no difference in fecundity between two successive mating females, three successive mating females, and three matings separated by two 7 d interval females (Fig. 2B).

### Number of Egg Clutches per Female and Number of Eggs per Clutch

**Fig. 3.**
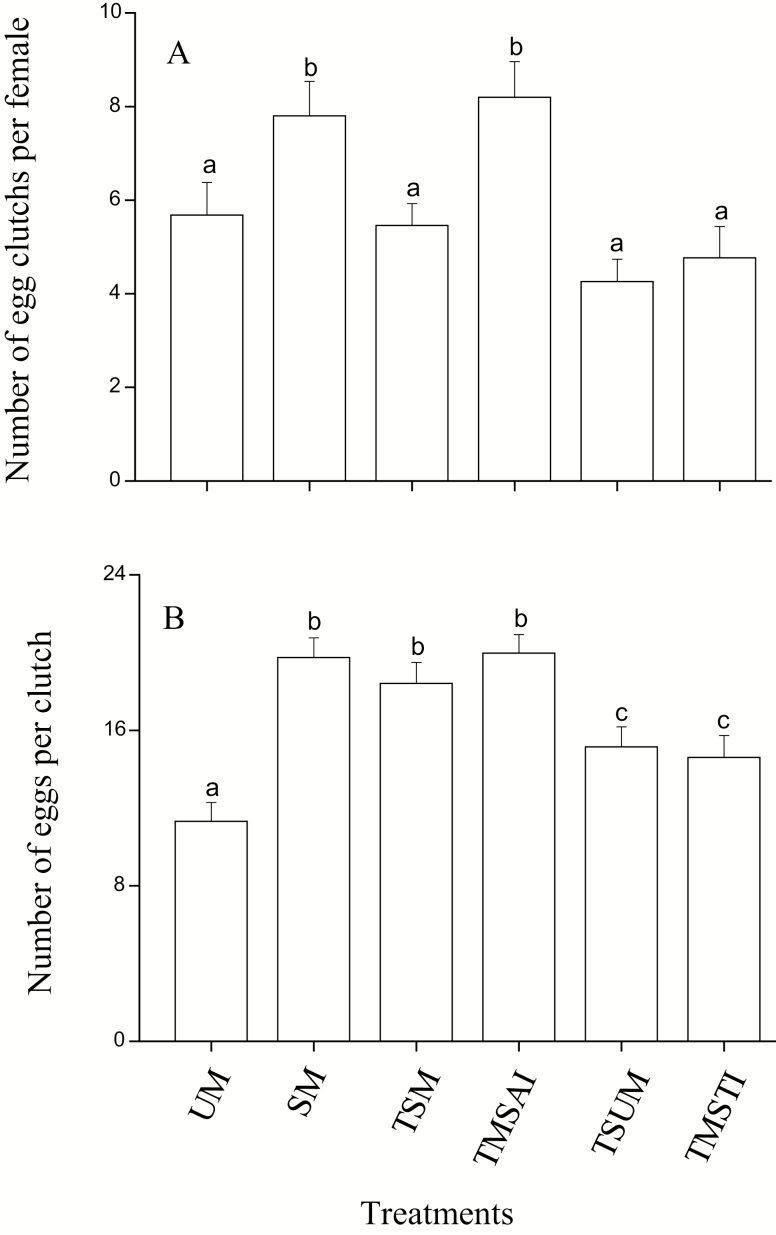
Number of egg clutches per female (A) and number of eggs per clutch (B). Error bars represent the SE. Bars with different letters are significantly different from each other at *P* < 0.05 by Fisher’s protected least significant test. UM: unmated females; SM: single mating; TSM: two successive matings; TMSAI: two matings separated by a 7 d interval; TSUM: three successive matings; TMSTI: three matings separated by two 7 d interval.

To further confirm that the reduction of fecundity was caused by decreased egg-laying capacity, we studied the number of egg clutches and the number of eggs per egg clutch produced by females during the entire oviposition period. Similar to fecundity, mating status influenced the number of egg clutches and the number of eggs per clutch during the entire oviposition period (ANOVA, number of egg clutches per female: *F* = 6.13, df = 5, 163, *P* < 0.001; number of eggs per clutch: *F* = 11.42, df = 5, 805, *P* < 0.001, Fig. 3). The number of egg clutches produced by females mated only once or two matings separated by a 7 d interval was greater than that of virgin females and two successive mating females, three successive mating females and three matings separated by two 7 d interval females (LSD test, once: *P* = 0.0017, 0.012, 0.0021, and 0.0013 respectively; two matings separated by a 7 d interval: *P* = 0.0042, 0.0034, 0.0012, and 0.0024 respectively, Fig. 3A), but there was no difference in the numbers of egg clutches among virgin females, two successive mating females, three successive mating females and three matings separated by two 7 d interval females (LSD test, *P* > 0.05 in all cases, Fig. 3A). The mean number of eggs per egg clutch produced by virgin females was significantly less than that produced by females that mated only once, twice (successive or interval) or three times (successive or interval) (LSD test, once and twice: *P* < 0.001; three times: *P* = 0.0092 and 0.026, Fig. 3B). The number of eggs per egg clutch produced by females mated twice was similar to that of females mated once (LSD test, *P* = 0.36 and 0.87); however, the number was greater than that of females mated three times (LSD test, three successive mating: *P* = 0.028 and *P* < 0.001; three matings separated by two 7 d interval: *P* = 0.012 and *P* < 0.001, Fig. 3B).

### Egg Hatch

**Fig. 4.**
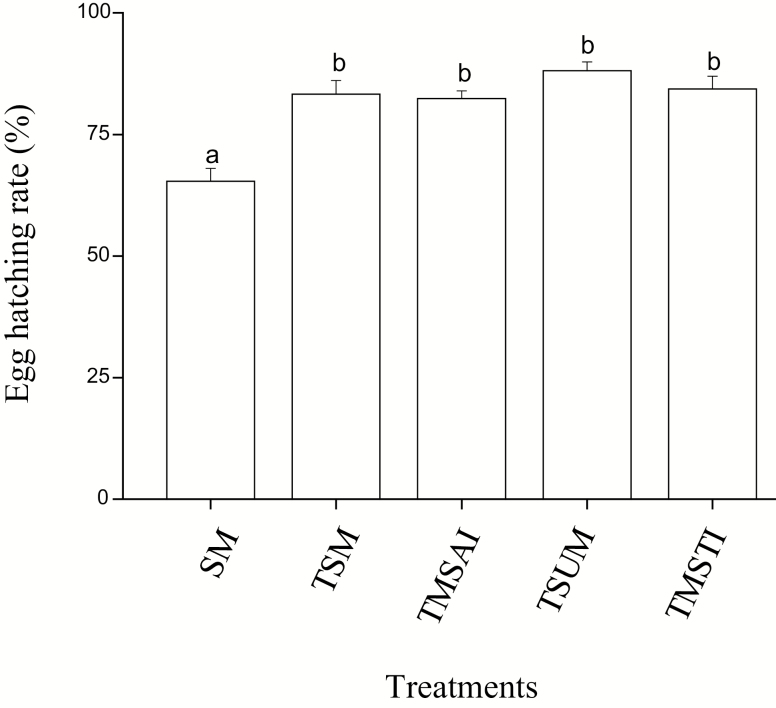
Egg hatching rate of the mating patterns. Error bars represent the SE. Bars with different letters are significantly different from each other at *P* < 0.05 by Fisher’s protected least significant test. UM: unmated females; SM: single mating; TSM: two successive matings; TMSAI: two matings separated by a 7 d interval; TSUM: three successive matings; TMSTI: three matings separated by two 7 d interval.

Mating status significantly influenced egg hatch (ANOVA, *F* = 6.13, df = 4, 129, *P* < 0.001, Fig. 4). The egg hatch of females mated once was less than that of females mated twice or three times (LSD test, two successive mating: *P* = 0.023; two matings separated by a 7 d interval: *P* = 0.012; three successive mating: *P* < 0.001; three matings separated by two 7 d interval: *P* = 0.0081), but there was no difference in egg hatch between females that mated twice or three times (LSD test, *P* > 0.05 in all cases).

## Discussion

Multiple mating in females is common but may involve fitness costs. It is generally thought that the costs of multiple mating should be offset by benefits that enhance female fitness (Hosken and Stockley 2003). Most studies evaluating the costs and benefits of multiple mating have used the ‘successive mating’ model (Arnqvist and Nilsson 2000). However, it is unclear whether there are differences in costs and benefits between interval multiple mating and successive multiple mating. Our results showed that two matings sufficed to fertilize the full egg load, and two matings separated by a 7 d interval was optimum for life span and fecundity in female *P. versicolora*. However, suboptimal mating frequency (two and three successive matings and three matings separated by two 7 d interval) reduced life span and fecundity.

Many studies demonstrate that multiple matings improve female lifespan and increase fecundity (e.g., Hosken amd Stockley 2003). The present study did not support these findings. The lifespan of females in two matings separated by a 7 d interval was similar to that of virgin females and once-mated females. However, two and three successive matings and three matings separated by two 7 d interval significantly reduced female longevity. Ridley (1988) proposed that multiple mating can increase female fecundity, but the act of mating itself may physically damage females and reduce longevity. In this study, the fecundity of females with two and three successive matings, as well as three matings separated by two 7 d interval, was significantly less than that of single mating females. Therefore, the reduction in longevity was not due to an increase in fecundity. The duration of egg-laying was also significantly shorter in females from two successive matings, three successive matings and three matings separated by two 7 d interval than that of single mating females. One possibility is that suboptimal multiple mating pattern (successive matings or excessive numbers of matings) causes physiological damage to *P. versicolora* and resulted in reduced longevity (Ridley 1988). A similar result was found in the windmill butterfly, *Atrophaneura alcinous*, where multiple mating caused physiological damage and reduced female longevity (Kawagoe et al. 2001).

Mating may increase predation risk, excessive energy consumption, injury during the mating process, and infection (Daly 1978). Therefore, the evolutionary benefits of multiple mating should outweigh those of single mating (Wang and Davis 2006). Arnqvist and Nilsson (2000) summarized 122 experimental studies on insect addressing the direct effects of multiple mating on female fitness in insects. Multiple-mated females of *Ctenocephalides felis* and *Galerucella birmanica* had significantly greater lifetime fecundity than single-mated females (Hsu and Wu 2000, Wang et al. 2018). However, our findings are not consistent with the results of these studies. Single mating females laid significantly more eggs than unmated females, but females in two successive matings, three successive matings and three matings separated by two 7 d interval had significantly decreased fecundity. Fecundity of two matings sepatated by a 7 d interval females was similar to single mating females, but significantly higher than virgin females, two successive matings females, three successive matings females, and three matings separated by two 7 d interval females. These results indicate that an optimized mating pattern (single mating or two matings separated by a 7 d interval) benefits female egg-laying. However, a suboptimal mating pattern (successive mating or excessive number of matings) will be less beneficial.

A direct benefit of multiple mating females in other species is that the female lifetime egg production generally increases with increasing mating frequency (Arnqvist and Nilsson 2000). However, our results are not consistent with these findings. Two and three successive matings and three matings separated by two 7 d interval treatments had a negative effect on *P. versicolora* fecundity. In theory, females with a shortened life span will have reduced fecundity. In *P. versicolora*, the number of lifetime eggs produced by females with two successive matings, three successive matings, and three matings separated by two 7 d interval were significantly lower than that of single mated females. In addition, the number of eggs laid by females with two and three successive matings and three matings separated by two 7 d interval were significantly lower than that of single mating females during the same oviposition period of 25 d after adult emergence. These results suggest that it is unlikely that shortened lifespan led to the reduction of fecundity in females with two and three successive matings and three matings separated by two 7 d interval, although the lifespan of females with two and three successive matings and three matings separated by two 7 d interval was shorter than that of single mating females. One possible explanation is that the physiological costs reducing the egg-laying capacity contributed the reduced fecundity of females with two and three successive matings and three matings separated by two 7 d interval because the mating act itself can damage female physiology (Daly 1978, Ridley 1988). In addition, the duration of mating increased with the number of matings, especially in two and three successive matings and three matings separated by two 7 d interval. Successive mating for long periods of time may be harmful to females because of the repeated inseminations, and the attachment of the mating plug itself might damage the females (Nilakhe 1977). This hypothesis is supported by fewer egg clutches in two successive matings, three successive matings and three matings separated by two 7 d interval and fewer eggs per egg clutch in three successive matings and three matings separated by two 7 d interval, compared to single mating. The number of egg clutches and number of eggs per egg clutch of two matings separated by a 7 d interval females were similar to single mating females. In contrast, females with two successive matings, three successive matings, and three matings separated by two 7 d interval produced fewer egg clutches than single mating females. Additionally, the number of eggs per clutch of females mated three times was less than that of the single mating females. These results suggest that a suboptimal mating pattern (excessive multiple mating or successive mating) reduces the ability of females to lay eggs. In summary, a suboptimal mating pattern creates a physiological cost to *P. versicolora* and reduces egg-laying.

Although unmated females of *P. versicolora* oviposited normal eggs, their eggs did not hatch, indicating that mating is necessary for *P. versicolora* to produce fertile eggs (data not shown). Egg hatchability was lower for females that mated only once than those mated multiple times; however, egg hatchability did not change in females that mated more than twice. This was consistent with the findings of *Nicrophorus vespilloides* Herbst, where females reached the highest level of fertility after two matings but additional matings did not affect fertility (House et al. 2008). The costs and benefits of mating for females interact to select for an optimal number of matings (Arnqvist and Nilsson 2000, Ronkainen et al. 2010). For *P. versicolora*, two matings sufficed to fertilize the full egg load. However, two successive matings, three successive matings, and three matings separated by two 7 d interval had negative effects on fecundity. This suggests that two matings separated by a 7 d interval is optimum for *P. versicolora* female fecundity. In fact, two matings separated by a 7 d interval is most likely to occur because beetles are solitary in nature. It is unlikely for a female to encounter a new male and then complete successive mating directly after the female has completed her first mating.

This study shows that multiple mating may not necessarily be beneficial to female reproductive fitness of *P. versicolora*. Rather, a suboptimal mating pattern can reduce longevity and fecundity. The shorter longevity and reduced fecundity of multiple mating females may result from the physiological cost of multiple mating. However, multiple mating can benefit egg hatch, as mating twice sufficed to fertilize the full egg load. Two matings separated by a 7 d interval was optimum for female fecundity in *P. versicolora*. The trade-offs between fecundity benefits and the costs of multiple mating may be related to selective pressures and the evolution of reproductive biology in this species.
